# Supplementary material for: How to Improve the Reliability of Aperiodic Parameter Estimates in M/EEG: A Method Comparison
Source: Psychophysiology. 2026 Mar 19;63(3):e70272. doi: 10.1111/psyp.70272 (PMC13000880; doi:10.1111/psyp.70272)
Supplement: Supplementary file 1 — Data S1: psyp70272‐sup‐0001‐Supinfo.pdf. [file PSYP-63-e70272-s001.pdf]

Supplementary Materials

Supplementary Analyses 1: Model Fit using Information Criterion and Mean Squared Error

Model performance was quantified using mean squared error (MSE) and Akaike's Information Criterion (AIC), both computed in log power space to meet the Gaussian error assumption. For each aperiodic estimation method, residuals were defined as the difference between the observed log power spectrum and the fitted aperiodic component; squared residuals therefore corresponded to the aperiodic-corrected power spectrum. These squared residuals were summed across frequencies to obtain a residual sum of squares (RSS).

The effective number of observations (n) corresponds to the number of frequency bins contributing to each model fit and therefore differs across methods in accordance with their modelling assumptions. Models that do not explicitly parameterize oscillatory peaks were evaluated either across the full frequency range (*full-reg*; n = 32) or with the alpha band (6–16 Hz) excluded to reduce contamination from oscillatory activity (*censor-reg*; n = 23). In contrast, models that explicitly model and separate periodic (oscillatory) components (*fooof0*, *fooof1*, *fooof3*) were evaluated across the full modelled frequency range (n = 32). Model complexity (k) is defined as the number of free parameters estimated by each approach. For models that do not explicitly parameterize oscillatory peaks (i.e., *full-reg* and *censor-reg*), k = 2 corresponds to an intercept and a slope. For *fooof* models, k reflects both aperiodic and oscillatory components. The aperiodic component contributes two parameters (offset/intercept and exponent/slope). Each oscillatory peak is described by three parameters: center frequency, amplitude, and bandwidth. Consequently, the *fooof* model without peaks (*fooof0*) has k = 2 (aperiodic parameters only), the *fooof* model with one peak (*fooof1*) has k = 5 (2 aperiodic + 3 peak parameters), and the *fooof* model with three peaks (*fooof3*) has k = 11 (2 aperiodic + 3 × 3 peak parameters).

Channel-level MSE was computed as: RSS/n, and AIC was calculated as: AIC = n · log(RSS/n) + 2k. AIC values were first computed at the channel level, then averaged within participants, and finally averaged across participants, yielding one group-level MSE and AIC estimate per experimental condition and estimation method.

Importantly, this analysis was performed only for spectra derived from the Fast Fourier Transform (FFT). Welch's method was not included because it involves segmenting the signal into overlapping windows and averaging their periodograms, which reduces the number of statistically independent frequency estimates. As a result, the effective sample size (n) cannot be defined in a straightforward fashion as the number of frequency bins, violating the assumptions underlying the AIC and MSE formulations used here.

The AIC-based comparison evaluates the *efficiency* of different aperiodic estimation approaches, defined as the trade-off between goodness of fit and model complexity, within the frequency domain that each method explicitly targets by design. Specifically, data-blind models (*full-reg* and *censor-reg*) are evaluated with respect to the frequency bins used to estimate the aperiodic slope (either the full frequency range or a reduced range with the alpha band excluded), whereas data-informed (*fooof0, fooof1, fooof3*) models are evaluated with respect to the fully modelled spectrum, including frequencies that may contain oscillatory structure. As a result, AIC comparisons are most directly interpretable within families of methods that share the same modelling goal and frequency coverage: comparisons among *fooof* models differing in the number of peaks, or comparisons between full and censored regression models that explicitly test the impact of excluding oscillatory frequencies. Comparisons across method families are informative at a more general level, indicating differences in efficiency given each method's modelling assumptions, but *should not* be interpreted as reflecting *absolute superiority* in explaining *identical* spectral data.

Accordingly, this analysis allows conclusions about whether added model flexibility, such as explicitly modelling oscillatory peaks, improves the efficiency ("fit") of aperiodic parameterization. It does not allow conclusions about the physiological reality of oscillations, the absolute accuracy of slope estimates, or which model best explains the entire power spectrum under identical constraints. In particular, a lower

AIC indicates a more parsimonious and generalizable description of the spectrum given the model's assumptions, not a *truer* representation of neural activity.

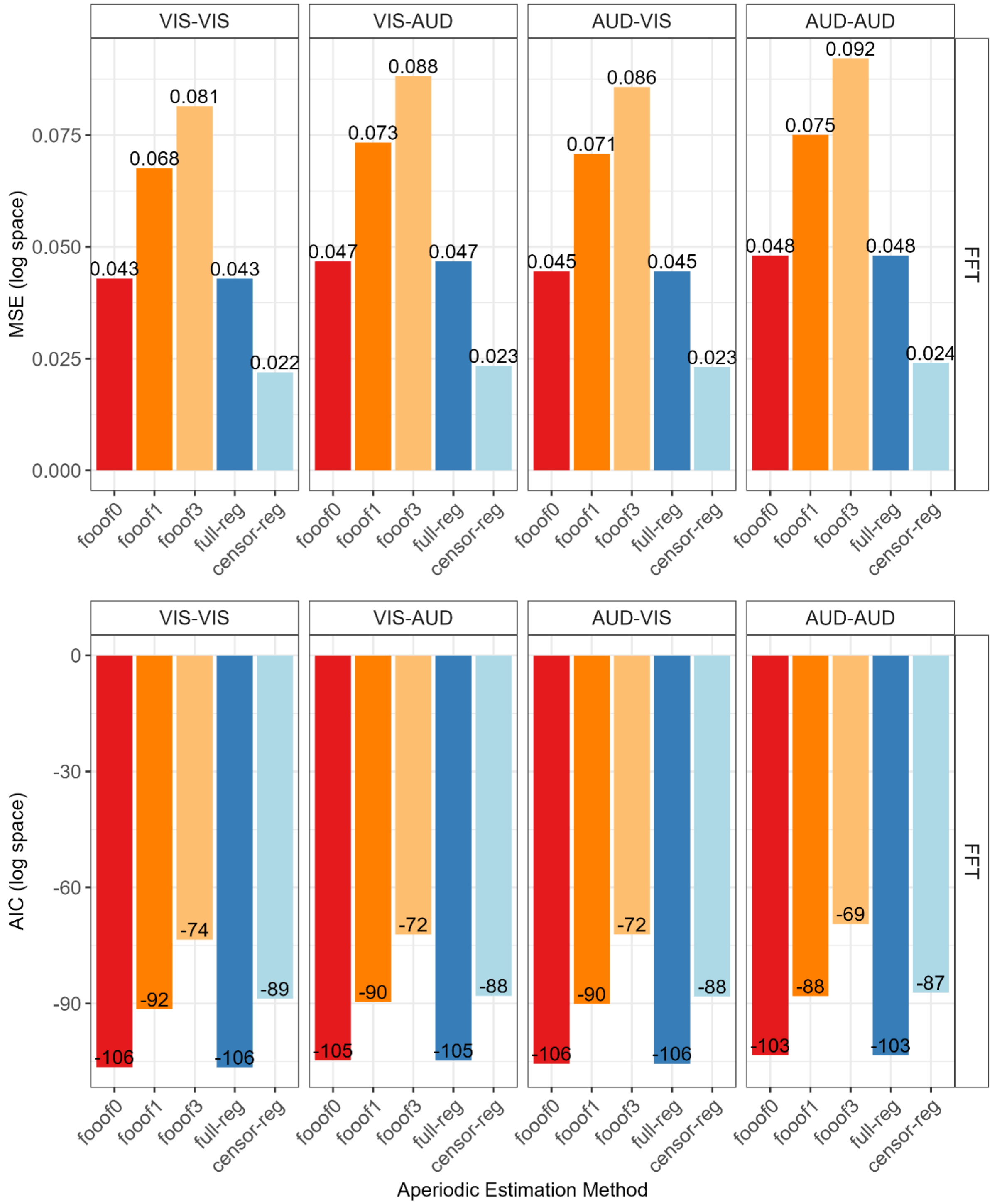

Figure S1. Comparison of Model-Fit Indices across Aperiodic Estimation Methods for the Stop-Signal Data set. The upper panel shows mean squared error (MSE) in log space, and the lower panel shows Akaike's Information Criterion (AIC) in log space, for each aperiodic estimation method (*fooof0, fooof1,* and *fooof3*, *fooof*-derived slope estimates with the corresponding number of modelled peaks; *full-reg,* full regression approach*; censor-reg,* censored regression approach). Bars represent aggregated values within each condition (for details, see the text). All values were computed from power spectra derived using the Fast Fourier Transform (FFT). Lower/more negative values indicate better model fit.

Across all four task versions in the stop signal data set (Figure S1), eyes open/closed in the resting state data set (Figure S2), and both spectral estimation approaches, model comparison based on AIC consistently favored simpler aperiodic parameterizations, with the *fooof0* and the *full-reg* models yielding the same and lowest AIC values. This indicates that both approaches provide a comparably efficient balance between model fit and complexity when evaluated across the full frequency range. Importantly, increasing the number of modelled peaks did not improve model efficiency: *fooof* models with one or three peaks showed higher MSE and were strongly penalized by AIC, demonstrating that added peak parameters did not yield sufficient gains in fit to justify their complexity. Censored regression model achieved the lowest MSE, indicating superior local approximation of the aperiodic background within non-oscillatory frequency ranges, and may therefore represent a practical choice when the primary goal is accurate slope estimation rather than full-spectrum efficiency.

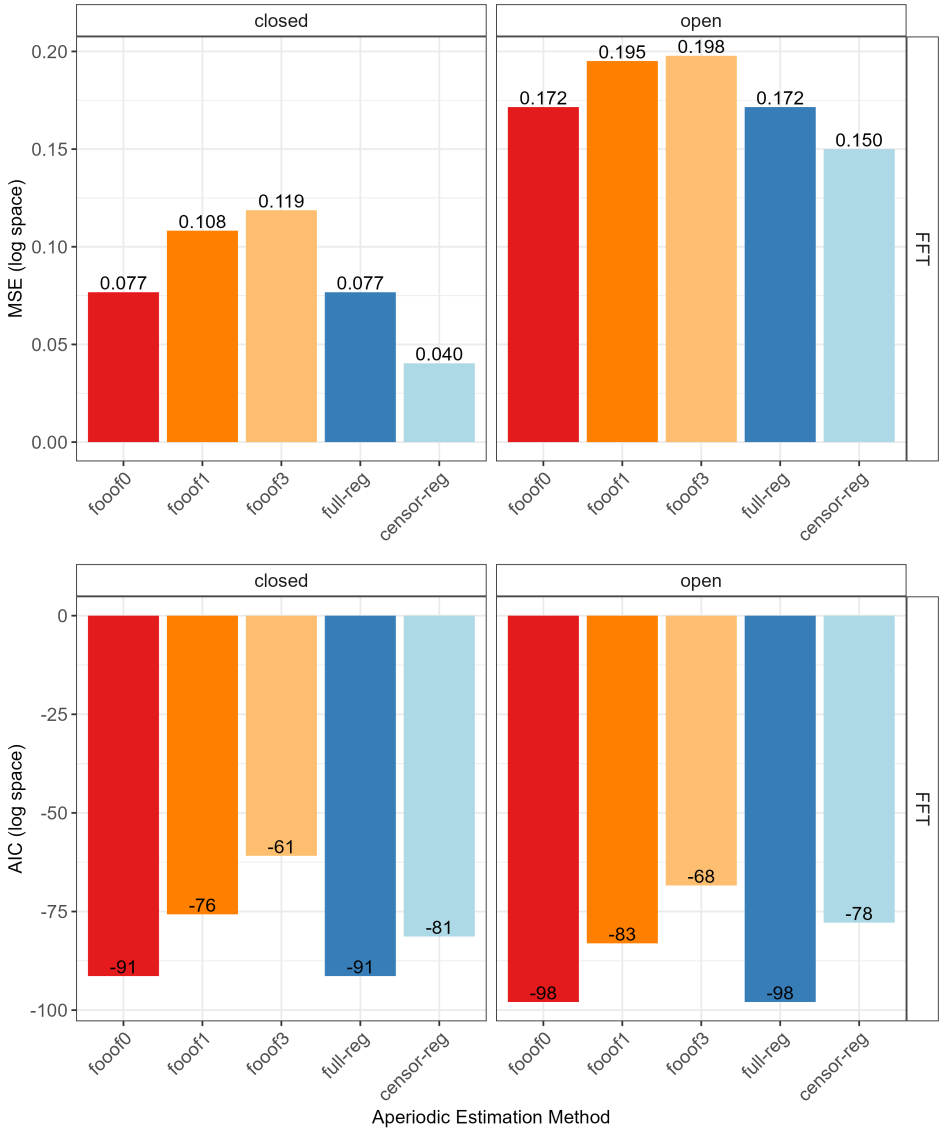

Figure S2. Comparison of Model-Fit Indices across Aperiodic Estimation Methods for the Resting State Data set. The upper panel shows mean squared error (MSE) in log space, and the lower panel shows Akaike's Information Criterion (AIC) in log space, for each aperiodic estimation method (*fooof0*, *fooof1*, and *fooof3*, *fooof*-derived slope estimates with the corresponding number of modelled peaks; *full-reg*, full regression approach; *censor-reg*, censored regression approach). Bars represent aggregated values within each condition (for details, see the text). All values were computed from power spectra derived using the Fast Fourier Transform (FFT). Lower/more negative values indicate better model fit.

Supplementary Analyses 2: Robustness of Censored Regression to Censoring Width

To evaluate the robustness of the censored regression approach to variations in the width of the censored frequency band, we conducted a simulation designed to quantify potential information loss due to censoring. We simulated 500 power spectra in log–log space as linear functions of frequency (2–33 Hz), with a ground-truth slope of −1.2 and an intercept of 1.0. To mimic realistic spectral structure, we added a Gaussian-shaped oscillatory peak centered at 10 Hz to each spectrum, along with random Gaussian noise to approximate measurement variability. For each simulated spectrum, the aperiodic slope was estimated using four approaches: (1) full regression across the entire 2–33 Hz range ("full"); (2) censored regression excluding 6–16 Hz, matching the procedure in the main manuscript ("truncated"); (3) censored regression excluding a wider frequency range than (2), i.e., 4–20 Hz ("aggressive"); (4) extreme censoring, using only the lowest and highest frequency points (i.e., two-point regression; "bookend"). Figure S3 presents the outcomes of this analysis.

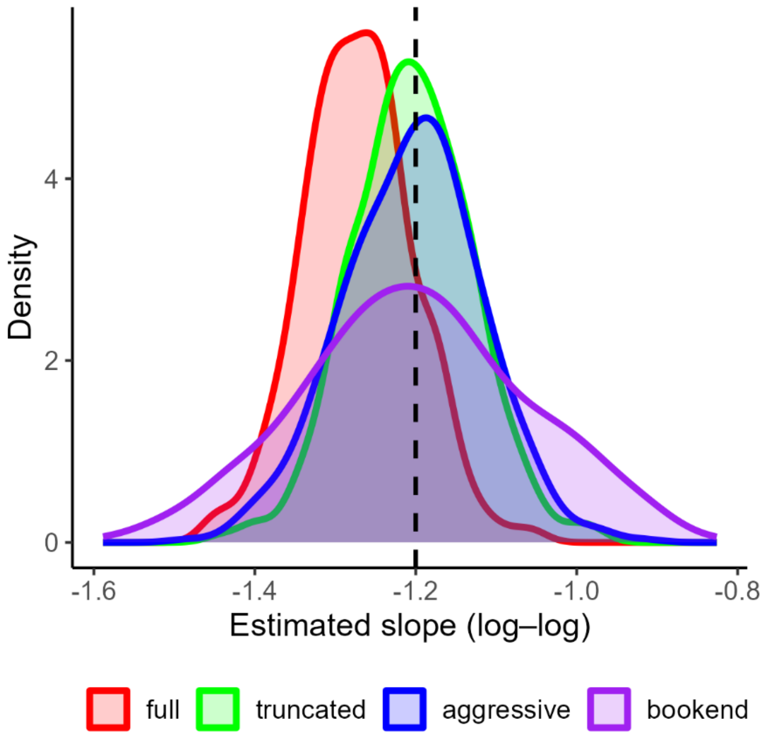

Figure S3. Robustness of Censored Regression to Censoring Width. Slope estimates were obtained using full regression ("full", red), standard censoring excluding 6–16 Hz ("truncated", green), aggressive censoring excluding 4–20 Hz ("aggressive"; blue), and extreme two-point regression ("bookend"; purple). The dashed vertical line indicates the true slope. For details, see the text.

The simulation demonstrates three key findings. First, full regression systematically misestimates the ground-truth slope, corroborating the main manuscript's conclusion that oscillatory peaks bias conventional regression approaches when the full frequency range is used. Second, both the standard (green) and aggressive (blue) censoring procedures yield virtually identical slope estimates, despite differing substantially in the number of excluded frequency bins. Importantly, the aggressive approach removes many frequency points that are not directly contaminated by the oscillatory peak, yet it produces estimates indistinguishable from the standard censoring procedure. This indicates that moderate over-censoring results in negligible loss of information for estimating the aperiodic slope. Finally, even extreme censoring (purple; based on only two frequency points) produces slope estimates centered around the true value.

However, this approach shows markedly greater variability, reflecting the increased sensitivity to noise when slope estimation relies on minimal data.

Together, these results demonstrate that censored regression is robust to reasonable variations in censoring width and that excluding oscillatory bands does not meaningfully distort recovery of the underlying aperiodic parameter.

Table S1.

Average Slope and Intercept Values across Participants and Electrodes for Each Estimation Approach after Outlier Removal

Spectral Decomposition	Aperiodic Estimation	Slope Mean	Slope SD	Slope Min	Slope Max	Intercept Mean	Intercept SD	Intercept Min	Intercept Max
**Resting State Data set**									
**N = 61, EEG Electrodes = 59. mean epochs = 45.45**									
FFT	fooof0	-1.06	0.53	-5.19	0.00	0.89	0.75	-1.39	6.81
FFT	fooof1	-1.11	0.66	-6.37	0.00	0.71	0.82	-1.89	7.11
FFT	fooof3	-1.20	0.65	-6.38	0.00	0.69	0.83	-2.10	7.14
FFT	reg-full	-1.06	0.53	-5.9	0.00	0.89	0.75	-1.39	6.81
FFT	reg-censor	-1.00	0.51	-5.06	0.00	0.78	0.71	-1.58	6.61
Welch	fooof0	-1.04	0.57	-5.68	0.00	0.82	0.76	-1.47	7.41
Welch	fooof1	-1.10	0.68	-6.21	0.00	0.64	0.81	-1.83	7.49
Welch	fooof3	-1.19	0.67	-6.91	0.00	0.62	0.81	-2.07	7.59
Welch	reg-full	-1.05	0.57	-5.36	0.00	0.82	0.76	-1.47	7.41
Welch	reg-censor	-0.98	0.51	-5.06	0.00	0.71	0.71	-1.58	7.20
**Stop-Signal Task Data set**									
**N = 34, EEG Electrodes = 32, mean epoch range 607-779**									
FFT	fooof0	-1.34	0.37	-2.49	-0.23	1.13	0.43	-0.26	2.55
FFT	fooof1	-1.35	0.55	-3.19	-0.03	0.86	0.63	-1.04	2.74
FFT	fooof3	-1.41	0.57	-3.21	-0.02	0.83	0.65	-1.14	2.71
FFT	reg-full	-1.34	0.37	-2.50	-0.23	1.13	0.43	-0.26	2.55
FFT	reg-censor	-1.28	0.37	-2.43	-0.15	1.01	0.44	-0.50	2.40
Welch	fooof0	-1.39	0.34	-2.49	-0.35	1.34	0.40	0.10	2.72
Welch	fooof1	-1.37	0.39	-2.62	-0.22	1.14	0.43	-0.28	2.57
Welch	fooof3	-1.42	0.38	-2.63	-0.25	1.14	0.43	-0.29	2.53
Welch	reg-full	-1.39	0.34	-2.49	-0.35	1.34	0.40	0.10	2.72
Welch	reg-censor	-1.33	0.33	-2.40	-0.29	1.23	0.39	-0.04	2.56

Note. Data were averaged across epochs, channels, and participants for each combination of Spectral Decomposition and Aperiodic Estimation. *FFT*, fast Fourier transform; *Welch*, Welch's method; *fooof0, fooof1,* and *fooof3, fooof*-derived slope estimates with the corresponding number of modelled peaks; *reg-full*, full regression approach; *reg-censor*, censored regression approach.

Table S2.

*Frequency of Improbable Values. **All Positive Slopes**, Bootstrap Analyses Ranked Results for Both Data sets*

Spectral Decomposition	Aperiodic Estimation	Probability of Being the Best	Mean Rank[a]
Resting State Data set – Eyes Open			
FFT	reg-censor	98.26	1.04
FFT	fooof0	1.76	2.48
FFT	reg-full	1.76	2.48
Welch	reg-censor	0.00	4.35
Welch	fooof0	0.00	5.35
Welch	reg-full	0.00	5.35
FFT	fooof1	0.00	7.28
FFT	fooof3	0.00	7.71
Welch	fooof3	0.00	9.00
Welch	fooof1	0.00	10.00
Resting State Data set – Eyes Closed			
FFT	fooof0	100	1.50
FFT	reg-full	100	1.50
FFT	reg-censor	0.00	3.00
Welch	fooof0	0.00	4.50
Welch	reg-censor	0.00	4.50
Welch	reg-full	0.00	6.00
FFT	fooof3	0.00	7.00
FFT	fooof1	0.00	8.00
Welch	fooof3	0.00	9.00
Welch	fooof1	0.00	10.00
Stop-Signal Task Data set			
Welch	fooof3	99.96	1.00
Welch	reg-censor	0.04	2.00
Welch	fooof1	0.00	3.00
Welch	fooof0	0.00	4.50
Welch	reg-full	0.00	4.50
FFT	reg-censor	0.00	6.00
FFT	fooof0	0.00	7.00
FFT	reg-full	0.00	7.99
FFT	fooof1	0.00	9.00
FFT	fooof3	0.00	10.0

Note. [a] smaller is better.

Table S3.

Frequency of Improbable Values. ***Positive Slopes Not Explained****, Bootstrap Analyses Ranked Results for Both Data sets*

Spectral Decomposition	Aperiodic Estimation	Probability of Being the Best	Mean Rank[a]
Resting State Data set – Eyes Open			
FFT	reg-censor	72.20	1.32
Welch	reg-censor	36.06	1.68
FFT	fooof0	0.00	3.50
FFT	reg-full	0.00	3.50
Welch	fooof0	0.00	5.50
Welch	reg-full	0.00	5.50
Welch	fooof3	0.00	7.74
FFT	fooof1	0.00	8.13
Welch	fooof1	0.00	8.13
FFT	fooof3	0.00	10.00
Resting State Data set – Eyes Closed			
FFT	reg-censor	90.58	1.10
Welch	reg-censor	9.58	1.91
FFT	fooof0	0.00	3.50
FFT	reg-full	0.00	3.50
Welch	fooof0	0.00	5.50
Welch	reg-full	0.00	5.50
Welch	fooof3	0.00	7.00
FFT	fooof3	0.00	8.02
FFT	fooof1	0.00	9.10
Welch	fooof1	0.00	9.87
Stop-Signal Task Data set			
FFT	reg-censor	100	1.50
Welch	reg-censor	100	1.50
Welch	fooof3	0.00	3.00
Welch	fooof0	0.00	4.53
Welch	reg-full	0.00	4.53
FFT	reg-full	0.00	6.28
FFT	fooof0	0.00	6.93
Welch	fooof1	0.00	7.73
FFT	fooof1	0.00	9.00
FFT	fooof3	0.00	10.0

Note. [a] smaller is better.

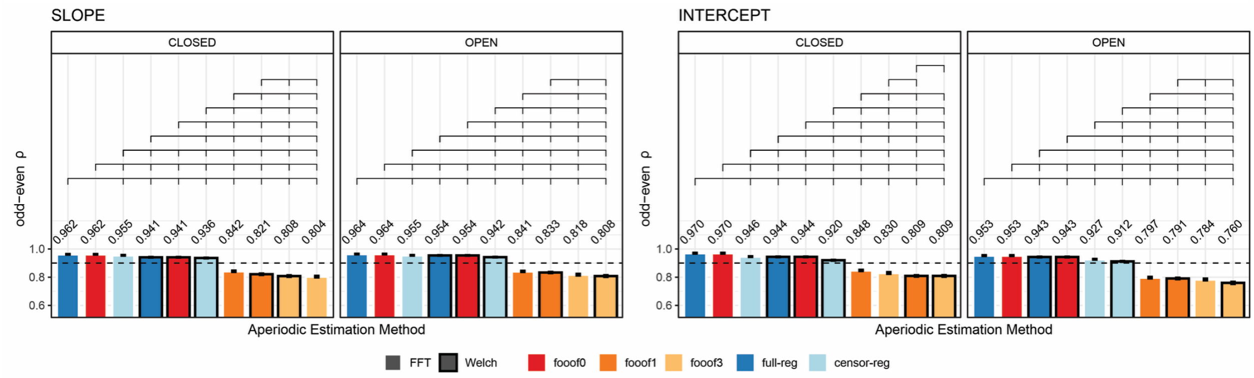

Figure S4. Internal consistency (odd-even reliability) after removing positive slopes for slope (left) and intercept (right) across aperiodic estimation methods, shown separately for eyes-closed and eyes-open during resting state. Within each subplot, bars are ordered from highest to lowest reliability. Brackets above the bars indicate statistically significant differences ($p < .05$ using permutation testing). Error bars reflect within-subject standard errors of the mean (SEM), computed using Morey's (2008) correction for repeated-measures designs. The horizontal dashed line represents a reliability threshold of .90, considered acceptable.

Table S4.

***Exponent** Odd-Even Reliability - Resting State Data set - Bootstrap Analyses Results*

Spectral Decomposition	Aperiodic Estimation	Probability of Being Best	Mean Rank[a]
Eyes Open – Positive Slopes Retained			
FFT	fooof0	100	1.5000
FFT	reg-full	100	1.5000
Welch	fooof0	0.00	3.5093
Welch	reg-full	0.00	3.5093
FFT	reg-censor	0.00	4.9814
Welch	reg-censor	0.00	6.0000
Welch	fooof1	0.00	7.4881
FFT	fooof1	0.00	7.5119
FFT	fooof3	0.00	9.0000
Welch	fooof3	0.00	10.0000
Eyes Closed - Positive Slopes Retained			
FFT	fooof0	100	1.5000
FFT	reg-full	100	1.5000
FFT	reg-censor	0.00	3.0000
Welch	fooof0	0.00	4.5002
Welch	reg-full	0.00	4.5002
Welch	reg-censor	0.00	5.9996
FFT	fooof1	0.00	7.2865
Welch	fooof1	0.00	7.7135
FFT	fooof3	0.00	9.3836
Welch	fooof3	0.00	9.6164
Eyes Open – Positive Slopes Removed			
FFT	fooof0	100.0	1.1795
FFT	reg-full	35.9	1.8205
FFT	reg-censor	0.00	3.4504
Welch	fooof0	0.00	4.2748
Welch	reg-full	0.00	4.2748
Welch	reg-censor	0.00	6.0000
FFT	fooof1	0.00	7.0314
Welch	fooof1	0.00	7.9711
FFT	fooof3	0.00	9.0265
Welch	fooof3	0.00	9.9710
Eyes Closed – Positive Slopes Removed			
FFT	fooof0	100	1.50
FFT	reg-full	100	1.50
FFT	reg-censor	0.00	3.00
Welch	fooof0	0.00	4.50
Welch	reg-full	0.00	4.50
Welch	reg-censor	0.00	6.00
FFT	fooof1	0.00	7.00
Welch	fooof1	0.00	8.00
Welch	fooof3	0.00	9.18
FFT	fooof3	0.00	9.82

Note. [a] smaller is better.

Table S5.

***Offset** Odd-Even Reliability - Resting State Data set – Bootstrap Analyses Results*

Spectral Decomposition	Aperiodic Estimation	Probability of Being Best	Mean Rank[a]
Eyes Open – Positive Slopes Retained			
FFT	fooof0	99.98	1.50
FFT	reg-full	99.98	1.50
Welch	fooof0	0.02	3.50
Welch	reg-full	0.02	3.50
FFT	reg-censor	0.00	5.00
Welch	reg-censor	0.00	6.00
Welch	fooof1	0.00	7.28
FFT	fooof1	0.00	7.73
FFT	fooof3	0.00	8.98
Welch	fooof3	0.00	10.00
Eyes Closed - Positive Slopes Retained			
FFT	fooof0	100.00	1.18
FFT	reg-full	35.98	1.82
Welch	fooof0	0.00	3.71
Welch	reg-full	0.00	3.71
FFT	reg-censor	0.00	4.58
Welch	reg-censor	0.00	6.00
FFT	fooof1	0.00	7.01
FFT	fooof3	0.00	7.99
Welch	fooof1	0.00	9.30
Welch	fooof3	0.00	9.70
Eyes Open – Positive Slopes Removed			
FFT	fooof0	100	1.50
FFT	reg-full	100	1.50
Welch	fooof0	0.00	3.50
Welch	reg-full	0.00	3.50
FFT	reg-censor	0.00	5.00
Welch	reg-censor	0.00	6.00
FFT	fooof1	0.00	7.12
Welch	fooof1	0.00	8.00
FFT	fooof3	0.00	8.87
Welch	fooof3	0.00	10.00
Eyes Closed– Positive Slopes Removed			
FFT	fooof0	100	1.50
FFT	reg-full	100	1.50
FFT	reg-censor	0.00	3.34
Welch	fooof0	0.00	4.33
Welch	reg-full	0.00	4.33
Welch	reg-censor	0.00	6.00
FFT	fooof1	0.00	7.00
FFT	fooof3	0.00	8.00
Welch	fooof1	0.00	9.46
Welch	fooof3	0.00	9.54

Note. [a] smaller is better.

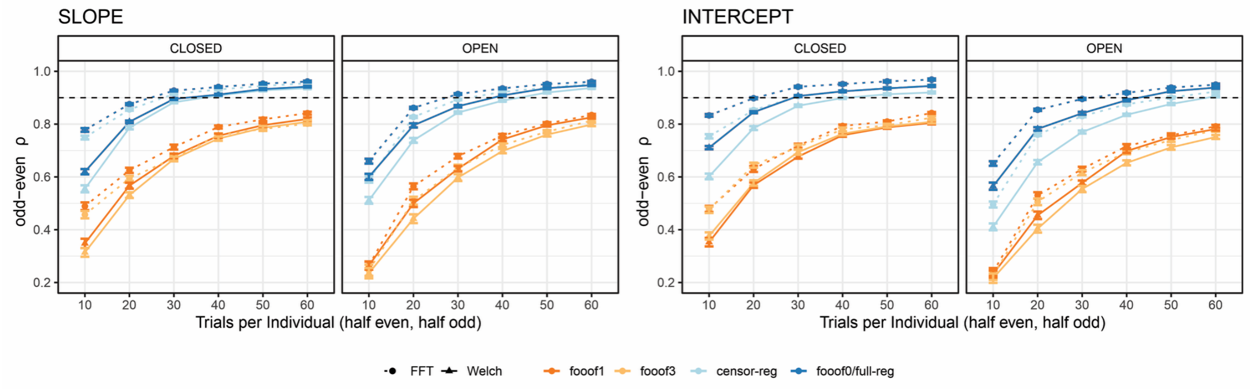

Figure S5. Internal consistency (odd-even reliability) as a function of trial count after removing positive slopes for slope (left) and intercept (right) across aperiodic estimation methods, shown separately for eyes-closed and eyes-open resting state. The horizontal dashed line represents a reliability threshold of .90, considered acceptable.

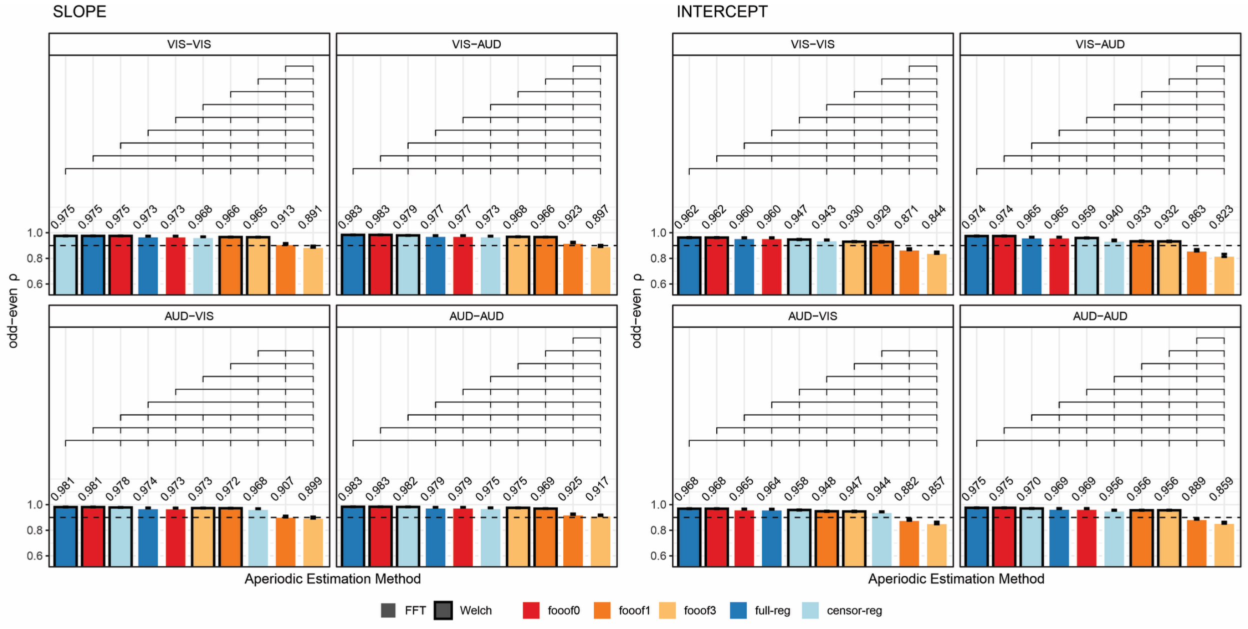

Figure S6. Internal consistency (odd-even reliability) after removing positive slopes for slope (left) and intercept (right) across aperiodic estimation methods, shown separately for each version of the stop-signal task. Within each subplot, bars are ordered from highest to lowest reliability. Brackets above the bars indicate statistically significant differences ($p < .05$, using permutation testing). Error bars reflect within-subject standard errors of the mean (SEM), computed using Morey's (2008) correction for repeated-measures designs. The horizontal dashed line represents a reliability threshold of .90, considered acceptable.

Table S6.

Exponent, Positive Slopes Retained, *Odd-Even Reliability - Stop Signal Data set – Bootstrap Analyses Results*

Spectral Decomposition	Aperiodic Estimation	Probability of Being the Best	Mean Rank[a]
Auditory-Auditory			
Welch	fooof0	94.62	1.56
Welch	reg-full	94.62	1.56
Welch	reg-censor	6.12	2.89
FFT	fooof0	0.00	4.28
FFT	reg-full	0.00	4.27
Welch	fooof3	0.00	6.32
FFT	reg-censor	0.00	6.67
Welch	fooof1	0.00	8.00
FFT	fooof1	0.00	9.00
FFT	fooof3	0.00	10.00
Auditory-Visual			
Welch	fooof0	99.98	1.50
Welch	reg-full	99.98	1.50
Welch	reg-censor	0.00	3.00
FFT	reg-full	0.00	4.80
FFT	fooof0	0.00	5.24
Welch	fooof3	0.00	5.67
Welch	fooof1	0.00	6.30
FFT	reg-censor	0.00	7.99
FFT	fooof1	0.00	9.00
FFT	fooof3	0.00	10.00
Visual-Auditory			
Welch	fooof0	100	1.50
Welch	reg-full	100	1.50
Welch	reg-censor	0.00	3.07
FFT	reg-full	0.00	4.09
FFT	fooof0	0.00	4.84
FFT	reg-censor	0.00	6.03
Welch	fooof3	0.00	7.08
Welch	fooof1	0.00	7.89
FFT	fooof1	0.00	9.00
FFT	fooof3	0.00	10.0
Visual-Visual			
Welch	fooof0	86.84	1.66
Welch	reg-full	86.84	1.66
Welch	reg-censor	12.82	2.96
FFT	reg-full	1.00	4.23
FFT	fooof0	0.84	4.50
FFT	reg-censor	0.00	6.22
Welch	fooof3	0.00	7.35
Welch	fooof1	0.00	7.43
FFT	fooof1	0.00	9.00
FFT	fooof3	0.00	10.00

Note. [a] smaller is better.

Table S7.

Exponent, Positive Slopes Removed, *Odd-Even Reliability - Stop Signal Data set – Bootstrap Analyses Results*

Spectral Decomposition	Aperiodic Estimation	Probability of Being the Best	Mean Rank[a]
Auditory-Auditory			
Welch	fooof0	94.12	1.56
Welch	reg-full	94.12	1.56
Welch	reg-censor	6.76	2.88
FFT	fooof0	0.00	4.30
FFT	reg-full	0.00	4.69
Welch	fooof3	0.00	6.40
FFT	reg-censor	0.00	6.59
Welch	fooof1	0.00	8.00
FFT	fooof1	0.00	9.00
FFT	fooof3	0.00	10.00
Auditory-Visual			
Welch	fooof0	99.88	1.50
Welch	reg-full	99.88	1.50
Welch	reg-censor	0.14	3.00
FFT	reg-full	0.00	4.66
FFT	fooof0	0.00	5.27
Welch	fooof3	0.00	5.42
Welch	fooof1	0.00	6.66
FFT	reg-censor	0.00	7.99
FFT	fooof1	0.00	9.01
FFT	fooof3	0.00	9.99
Visual-Auditory			
Welch	fooof0	100	1.50
Welch	reg-full	100	1.50
Welch	reg-censor	0.00	3.05
FFT	reg-full	0.00	4.03
FFT	fooof0	0.00	4.94
FFT	reg-censor	0.00	6.01
Welch	fooof3	0.00	7.04
Welch	fooof1	0.00	7.95
FFT	fooof1	0.00	9.00
FFT	fooof3	0.00	10.00
Visual-Visual			
Welch	fooof0	82.88	1.69
Welch	reg-full	82.88	1.69
Welch	reg-censor	18.04	2.74
FFT	fooof0	0.20	4.34
FFT	reg-full	0.40	4.55
FFT	reg-censor	0.00	6.28
Welch	fooof1	0.00	7.04
Welch	fooof3	0.00	7.95
FFT	fooof1	0.00	9.00
FFT	fooof3	0.00	10.00

Note. [a] smaller is better.

Table S8.

Offset, Positive Slopes Retained, *Odd-Even Reliability - Stop Signal Data set – Bootstrap Analyses Results*

Spectral Decomposition	Aperiodic Estimation	Probability of Being the Best	Mean Rank[a]
Auditory-Auditory			
Welch	fooof0	100	1.50
Welch	reg-full	100	1.50
FFT	reg-full	0.00	3.58
FFT	fooof0	0.00	4.12
Welch	reg-censor	0.00	4.30
Welch	fooof3	0.00	6.93
Welch	fooof1	0.00	6.96
FFT	reg-censor	0.00	7.12
FFT	fooof1	0.00	9.00
FFT	fooof3	0.00	10.00
Auditory-Visual			
Welch	fooof0	99.74	1.50
Welch	reg-full	99.74	1.50
FFT	fooof0	0.00	3.07
FFT	reg-full	0.00	3.96
Welch	reg-censor	0.00	3.96
FFT	fooof1	0.00	6.56
Welch	fooof3	0.00	6.79
Welch	reg-censor	0.00	7.65
FFT	fooof1	0.00	9.00
FFT	fooof3	0.00	10.0
Visual-Auditory			
Welch	fooof0	100	1.50
Welch	reg-full	100	1.50
FFT	fooof0	0.00	3.33
FFT	reg-full	0.00	3.67
Welch	reg-censor	0.00	5.00
FFT	reg-censor	0.00	6.02
Welch	fooof1	0.00	7.44
Welch	fooof3	0.00	7.54
FFT	fooof1	0.00	9.00
FFT	fooof3	0.00	10.00
Visual-Visual			
FFT	fooof0	36.50	2.32
FFT	reg-full	27.88	2.50
Welch	fooof0	44.34	2.59
Welch	reg-full	44.34	2.59
Welch	reg-censor	0.00	5.14
FFT	reg-censor	0.00	5.86
Welch	fooof1	0.00	7.19
Welch	fooof3	0.00	7.81
FFT	fooof1	0.00	9.00
FFT	fooof3	0.00	10.00

Note. [a] smaller is better.

Table S9.

Offset, Positive Slopes Removed, *Odd-Even Reliability - Stop Signal Data set – Bootstrap Analyses Results*

Spectral Decomposition	Aperiodic Estimation	Probability of Being the Best	Mean Rank[a]
Auditory-Auditory			
Welch	fooof0	100	1.50
Welch	reg-full	100	1.50
Welch	reg-censor	0.00	3.79
FFT	fooof0	0.00	3.94
FFT	reg-full	0.00	4.27
Welch	fooof1	0.00	6.63
FFT	Rec-censor	0.00	7.04
Welch	fooof3	0.00	7.33
FFT	fooof1	0.00	9.00
FFT	fooof3	0.00	10.00
Auditory-Visual			
Welch	fooof0	96.10	1.57
Welch	reg-full	96.10	1.57
FFT	fooof0	3.98	2.97
FFT	reg-full	0.20	3.92
Welch	reg-censor	0.00	4.98
WElch	fooof1	0.00	6.31
Welch	fooof3	0.00	6.86
FFT	reg-censor	0.00	7.83
FFT	fooof1	0.00	9.00
FFT	fooof3	0.00	10.00
Visual-Auditory			
Welch	fooof0	100	1.50
Welch	reg-full	100	1.50
FFT	fooof0	0.00	3.49
FFT	reg-full	0.00	3.51
Welch	reg-censor	0.00	5.00
FFT	reg-censor	0.00	6.14
Welch	fooof1	0.00	7.35
Welch	fooof3	0.00	7.51
FFT	fooof1	0.00	9.00
FFT	fooof3	0.00	10.00
Visual-Visual			
Welch	fooof0	87.32.	1.73
Welch	reg-full	87.23	1.73
FFT	reg-full	10.62	2.90
FFT	fooof0	3.74	3.64
Welch	reg-censor	0.00	5.03
FFT	reg-censor	0.00	5.97
Welch	fooof3	0.00	7.30
Welch	fooof1	0.00	7.70
FFT	fooof1	0.00	9.00
FFT	fooof3	0.00	10.00

Note. [a] smaller is better.

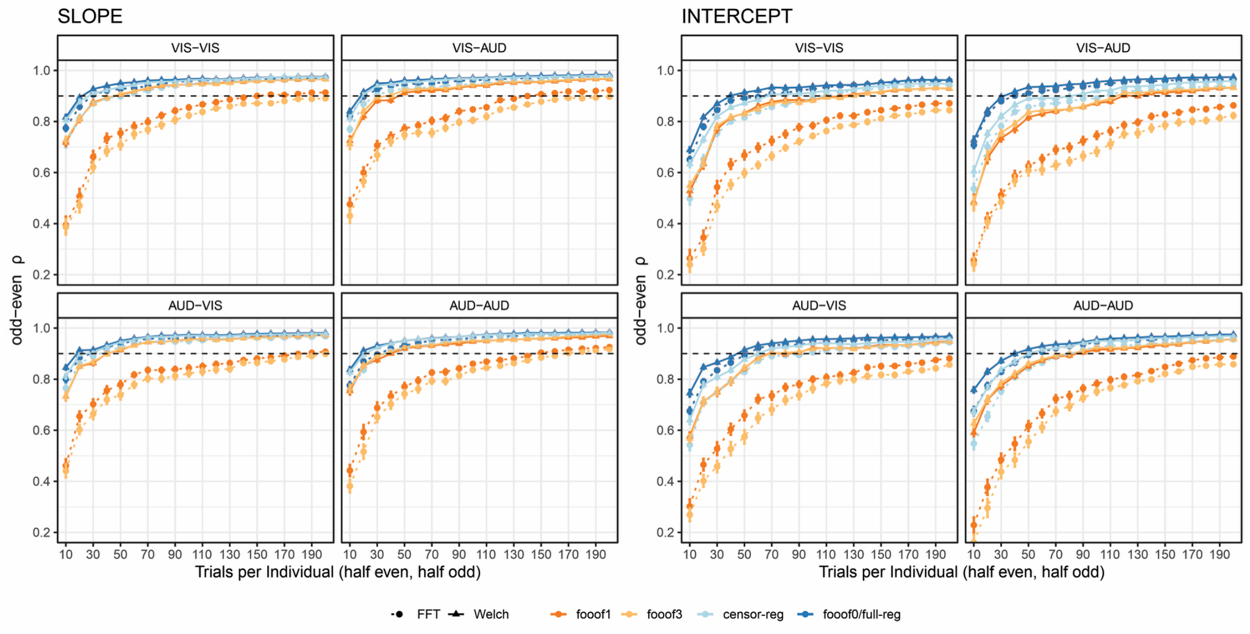

Figure S7. Internal consistency (odd-even reliability) as a function of trial count after removing positive slopes for slope (left) and intercept (right) across aperiodic estimation methods, shown separately for each version of the stop-signal task. The dashed horizontal line represents a reliability threshold of .90, considered acceptable.

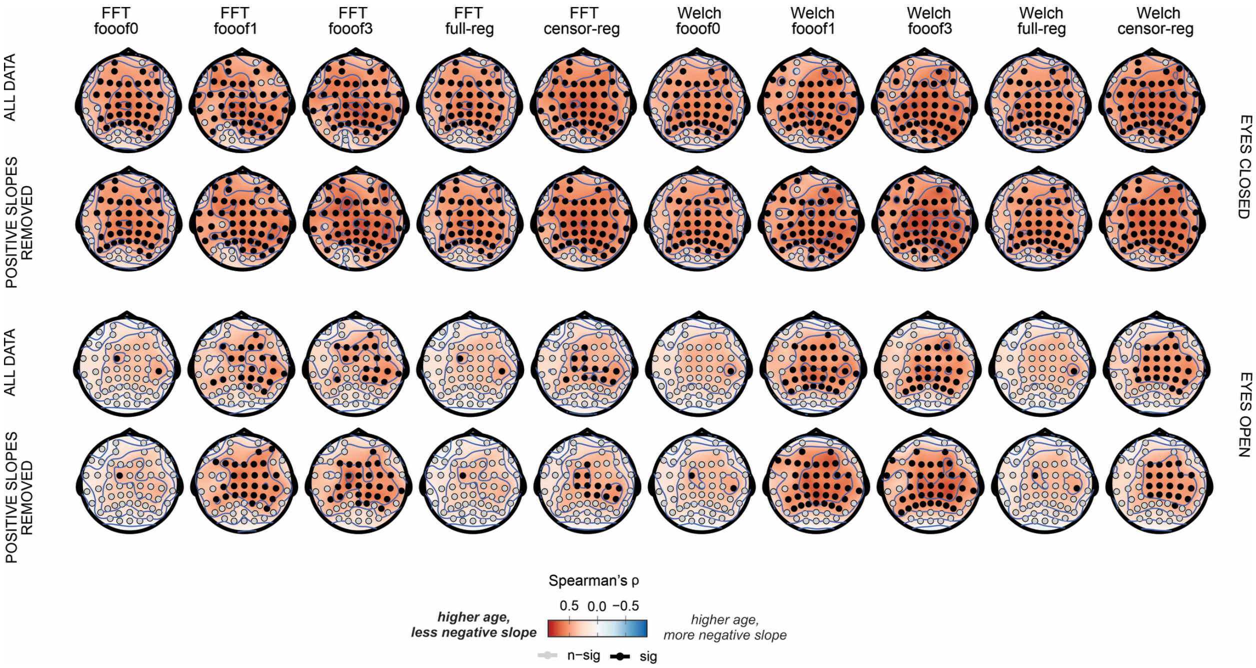

Figure S8. Electrode-Level Correlations Between Age and Slope in the Resting-State Data set. Spearman's rho correlations between age and slope at each electrode across aperiodic estimation methods for eyes-closed (top) and eyes-open (bottom) resting-state conditions, shown for the full slope data set (upper) and after removing positive slopes (lower). Statistical significance ($p < .05$) was assessed using a permutation test. *FFT*, fast Fourier transform; *Welch*, Welch's method; *fooof0, fooof1, and fooof3*, *fooof*-derived slope estimates with the corresponding number of modelled peaks; *full-reg*, full regression approach; *censor-reg*, censored regression approach.

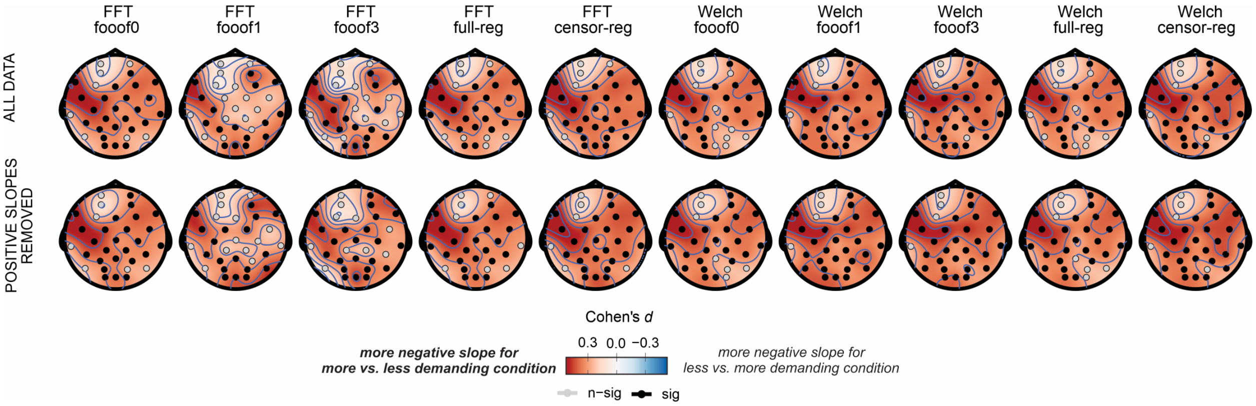

Figure S9. Electrode-Level Experimental Effects in the Stop-Signal Data set. Effect sizes (Cohen's *d*) for slope comparisons between AUD-VIS and VIS-VIS at each electrode across aperiodic estimation methods, presented for the full slope data set (top) and after removing positive slopes (bottom). Positive values indicate a more negative slope for AUD-VIS relative to VIS-VIS. Statistical significance ($p < .05$) was assessed using a permutation test.
